# Ghrelin, Des-Acyl Ghrelin, and Obestatin: Regulatory Roles on the Gastrointestinal Motility

**DOI:** 10.1155/2010/305192

**Published:** 2010-03-15

**Authors:** Mineko Fujimiya, Akihiro Asakawa, Koji Ataka, Chih-Yen Chen, Ikuo Kato, Akio Inui

**Affiliations:** ^1^Department of Anatomy, Sapporo Medical University School of Medicine, Sapporo 060-8556, Japan; ^2^Department of Behavioral Medicine, Kagoshima University Graduate School of Medical and Dental Sciences, Kagoshima 890-8520, Japan; ^3^Research Institute, Taiko Pharmaceutical Co., Ltd., Osaka 564-0032, Japan; ^4^Department of Internal Medicine, Faculty of Medicine, National Yang-Ming University School of Medicine, Taipei 112, Taiwan; ^5^Department of Bioorganic Chemistry, Faculty of Pharmaceutical Sciences, Hokuriku University, Kanazawa 920-1181, Japan

## Abstract

Ghrelin, des-acyl ghrelin, and obestatin are derived from a common prohormone, preproghrelin by posttranslational processing, originating from endocrine cells in the stomach. To examine the regulatory roles of these peptides, we applied the manometric measurement of gastrointestinal motility in freely moving conscious rat or mouse model. Ghrelin exerts stimulatory effects on the motility of antrum and duodenum in both fed and fasted state of animals. Des-acyl ghrelin exerts inhibitory effects on the motility of antrum but not on the motility of duodenum in the fasted state of animals. Obestatin exerts inhibitory effects on the motility of antrum and duodenum in the fed state but not in the fasted state of animals. NPY Y2 and Y4 receptors in the brain may mediate the action of ghrelin, CRF type 2 receptor in the brain may mediate the action of des-acyl ghrelin, whereas CRF type 1 and type 2 receptors in the brain may mediate the action of obestatin. Vagal afferent pathways might be involved in the action of ghrelin, but not involved in the action of des-acyl ghrelin, whereas vagal afferent pathways might be partially involved in the action of obestatin.

## 1. Introduction

Ghrelin, des-acyl ghrelin, and obestatin are derived from a prohormone, preproghrelin by posttranslational processing. Ghrelin was first identified as endogenous ligand for growth hormone secretagogue receptors (GHS-R) with O-n-octanoyl acid modification at serine 3 position [1]. Des-acyl ghrelin, on the other hand, has the same amino acid sequence with no O-n-octanoyl acid modification [1]. Obestatin was found by a bioinformatics approach to be encoded by preproghrelin [2]. Obestatin was initially reported to be endogenous ligand for orphan G protein-coupled receptor GPR39 [2]; however recent studies have found no specific binding of obestatin to various types of GPR39-expressing cells [3–5]. Ghrelin is a potent stimulator of food intake and gastrointestinal motility [6], while des-acyl ghrelin exerts opposite effects on food intake and gastrointestinal motility [7]. The effects of obestatin on food intake and gastrointestinal motility have been controversial [8–13].

Recently we developed conscious rat and mouse models to measure physiological fed and fasted motor activities in the gastrointestinal tracts [14–18]. By using these models we succeeded to examine the effects of ghrelin, des-acyl ghrelin, and obestatin on gastroduodenal motility and involvement of hypothalamic peptides mediating the action of these peptides. In this review, we overview the different effects of ghrelin, des-acyl ghrelin, and obestatin on the upper gastrointestinal motility with special attention being paid to brain-gut interactions.

## 2. Localization of Ghrelin, Des-Acyl Ghrelin, and Obestatin in the Rat Stomach

The localization of ghrelin in the stomach has been studied in various animals by using the specific antibody for ghrelin [19, 20]; however, the localization of des-acyl ghrelin in the stomach has been scarcely examined. We developed antibodies specific for ghrelin (anti-rat octanoyl ghrelin (1-15)-cys-KLH serum) and for des-acyl ghrelin (anti-rat des-octanoyl ghrelin (1-15)-cis-KLH serum) and successfully detected the different localization of ghrelin and des-acyl ghrelin in the rat stomach [21].

**Figure 1:**
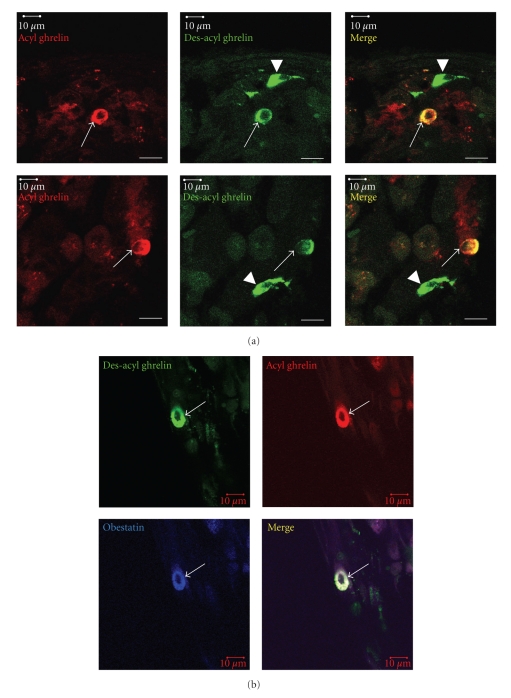
Localization of ghrelin, des-acyl ghrelin and obestatin in the rat stomach. (a) Immunofluorescence double staining for acyl ghrelin- (red) and des-acyl ghrelin-positive (green) reaction in the antral mucosa of rat stomach. Acyl ghrelin-positive reaction and des-acyl ghrelin-positive reaction are colocalized in closed-type cells (arrows), whereas des-acyl ghrelin-positive reaction is localized in open-type cells (arrowheads). (b) Immunofluorescence triple staining for des-acyl ghrelin (green), acyl ghrelin (red) and obestatin (blue) in the antral mucosa of rat stomach. Three peptides are colocalized in the closed-type cells (arrows). Bars = 10 *μ*m.

Both ghrelin- and des-acyl ghrelin-immunoreactive cells were distributed in the oxyntic and antral mucosa of the rat stomach, with higher density in the antral mucosa than oxyntic mucosa. Immunofluorescence double staining showed that ghrelin- and des-acyl ghrelin-positive reactions overlapped in closed-type round cells, whereas des-acyl ghrelin-positive reaction was found in open-type cells in which ghrelin was negative (Figure 1(a)). Ghrelin/des-acyl ghrelin-positive closed-type cells contain obestatin (Figure 1(b)); on the other hand des-acyl ghrelin-positive open-type cells contain somatostatin [21].

The characteristic features of open-type cells that contain des-acyl ghrelin and closed-type cells that contain ghrelin indicate that they may respond differently to intraluminal factors. It is highly possible that open-type cells may react to luminal stimuli more than closed-type cells. Therefore we investigated the effects of different intragastric pH levels on the release of ghrelin and that of des-acyl ghrelin from the ex vivo perfused rat stomach [21]. In a preliminary study we measured the intragastric pH levels in the fasting and fed states of rats and found that intragastric pH in the fasting state was pH 4, whereas that in the fed state was pH 2 [16]. Our results showed that the release of ghrelin was not affected by intragastric pH, whereas the release of des-acyl ghrelin was increased at intragastric pH 2 compared to that at intragastric pH 4 [21]. This result suggests that des-acyl ghrelin-containing cells may sense the intragastric pH via their cytoplasmic processes and release the peptide in accordance with the lower intragastric pH. The fact that the release of des-acyl ghrelin is stimulated by lower intragastric pH seems reasonable because des-acyl ghrelin may act as a satiety signal [6, 7] in the fed state of animals. 

## 3. Manometric Measurement of Gastrointestinal Motility in Conscious Mice and Rats

We developed freely moving conscious animal model to measure the gastrointestinal motility in rats [15] and mice [18]. This model permits the measurement of gastrointestinal motility in animals in the physiological fed and fasted states by a manometric method [15, 18]. In the fasted state, the cyclic changes of pressure waves were detected in both antrum and duodenum, including the quiescence period during which relatively low amplitude contractions occur (phase I-like contractions), followed by a grouping of strong contractions (phase III-like contractions). The frequencies of phase III-like contractions in the fasted motility in the antrum and duodenum in mice (6.0 ± 0.2/h and 6.0 ± 0.3/h, resp.) were significantly (*P* < .05) higher than those in rats (5.3 ± 0.5/h, 5.6 ± 0.8/h, resp.) [15, 18]. After food intake, such fasted motor pattern was disrupted and replaced by a fed motor pattern, which consisted of irregular contractions of high frequency.

## 4. Ghrelin and Gastroduodenal Motility

**Figure 2:**
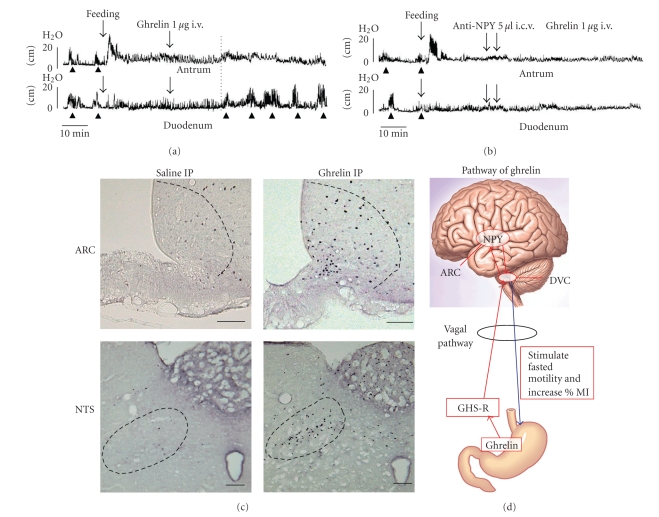
Effects of ghrelin on the gastroduodenal motility. (a) Effects of i.v. injection of ghrelin on the fed motor activity of the antrum and duodenum. I.v. injection of ghrelin induces the fasted pattern in the duodenum and increases the motor activity in the antrum. (b) I.c.v. injection NPY antiserum completely blocks the effect of i.v. injection of ghrelin. (c) The density of c*-Fos*-positive cells in the arcuate nucleus (ARC) and NTS is increased by i.p. injection of ghrelin compared to saline-injected control. (d) Summary diagram of the effects of ghrelin on the gastroduodenal motility and brain mechanism mediating its action.

Intracerebroventricular (i.c.v.) and intravenous (i.v.) injection of ghrelin stimulated the % motor index (%MI) in the antrum and induced the fasted motor activity in the duodenum when given in the fed state of animals [16, 18] (Figure 2(a)). I.c.v. and i.v. injection of ghrelin increased the frequency of phase III-like contractions in both antrum and duodenum when given in the fasted state of animals [16]. The effects of i.v. injection of ghrelin on gastroduodenal motility were blocked by i.v. injection of GHS-R antagonist but not by i.c.v. injection of GHS-R antagonist [16]. Immunoneutralization of NPY in the brain blocked the stimulatory effects of ghrelin on the gastroduodenal motility [16] (Figure 2(b)). These results indicate that ghrelin released from the stomach may act on the ghrelin receptor on vagal afferent nerve terminals and NPY neurons in the brain may mediate the action of ghrelin on the gastroduodenal motility (Figures 2(c) and 2(d)). Our previous study showed that immunoneutralization of NPY in the brain completely blocked the phase III-like contractions in the duodenum of normal rats, and Y2 and Y4 receptor agonists induced the phase III-like contractions in the duodenum when given in the fed state of animals [15]. Combined together, in normal animals ghrelin may stimulate gastroduodenal motility by activating the GHS-R on vagal afferent nerve terminals and affect NPY neurons in the hypothalamus, and Y2 and/or Y4 receptors in the brain may mediate the action of ghrelin (Figure 2(d), Table 1). Once the brain mechanism is eliminated by truncal vagotomy, ghrelin might be primarily involved in the regulation of fasted molility through GHS-R on the stomach and duodenum [16].

Human ghrelin has a structural resemblance to human motilin, and human ghrelin receptors exhibit a 50% identity with human motilin receptors [22]. Therefore the role of ghrelin in the gastrointestinal motility is comparable with that of motilin [23, 24]. Motilin originates from the endocrine cells in the duodenum [23], while ghrelin originates from the endocrine cells in the stomach [20]; both of them are involved in the regulation of phase III contractions in the gastrointestinal tracts. Motilin induces fasted motility in the stomach and duodenum when it is given peripherally but not when given centrally [24, 25], while ghrelin induces fasted motility in the duodenum when it is given both peripherally and centrally [16]. Since it is known that gastric acidification modulates the action of motilin [26], we examined the relationship between the effects of ghrelin on gastroduodenal motility and intragastric pH. The results showed that within 30 minutes after feeding low intragastric pH (pH 2.5 ± 0.2) inhibited the effects i.v. injected ghrelin on gastroduodenal motility, and that this effect was reversed by an increase of intragastric pH (pH 5.4 ± 0.6) within 60 minutes after feeding, or by pretreatment of famotidine (intragastric pH 6.0–6.7) [16]. These results suggest that the sensitivity of the GHS-R in the gastrointestinal tract might be inhibited by low intragastric pH.

## 5. Des-Acyl Ghrelin and Gastroduodenal Motility

Central and peripheral administration of des-acyl ghrelin has been shown to significantly decrease food intake in food-deprived mice and decrease gastric emptying [6]. Transgenic mice with overexpression of the des-acyl ghrelin gene exhibited a decrease in body weight, food intake, and fat mass weight accompanied by moderately decreased linear growth compared with their nontransgenic littermates [6]. In rats, des-acyl ghrelin injected intraperitoneally (i.p.) effectively decreased food intake in food-deprived rats and decreased the dark-phase food intake in free-feeding rats but failed to decrease the light-phase food intake in free-feeding rats [7].

**Figure 3:**
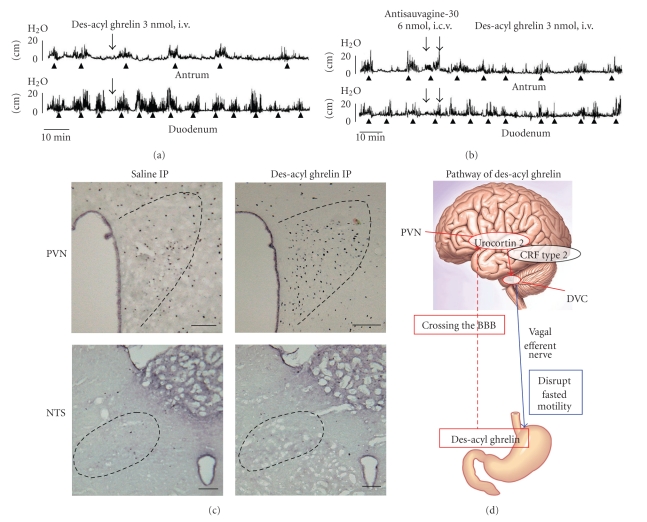
Effects of des-acyl ghrelin on the gastroduodenal motility. (a) Effects of i.v. injection of des-acyl ghrelin on the fasted motor activities of the antrum and duodenum. I.v. injection of des-acyl ghrelin decreases the frequency of phase III-like contractions in the antrum but not in the duodenum. (b) The decreased frequency of phase III-like contractions induced by i.v. injection of des-acyl ghrelin is restored to normal in pretreatment of i.c.v. injection of the selective CRF type 2 receptor antagonist antisauvagine-30. (c) The density of c-*Fos*-positive cells in the PVN is increased by i.p. injection of des-acyl ghrelin compared to saline-injected control, whereas that in the NTS is not altered. (d) Summary diagram of the effects of des-acyl ghrelin on the gastroduodenal motility and brain mechanism mediating its action.

I.c.v. and i.v. injections of des-acyl ghrelin disrupted fasted motility in the antrum but not in the duodenum [7] (Figure 3(a)). The frequencies of fasted motility in the antrum were decreased to 58.9% and 54.5% by des-acyl ghrelin injcted i.c.v. and i.v., respectively, [7]. However i.c.v. and i.v. injections of des-acyl ghrelin did not alter fed motor activity in both the antrum and duodenum [7]. These data indicate that the dominant role of exogenous des-acyl ghrelin affects fasted motility in the antrum but not in the duodenum. The results showed that capsaicin treatment did not alter the disruptive effect of i.v. injection of des-acyl ghrelin on fasted motility in the antrum [7]. These results were consistent with electrophysiological studies, which showed that peripheral administration of ghrelin suppressed firing of the vagal afferent pathways, whereas des-acyl ghrelin had no effect on vagal afferent pathways [27]. Difference in the involvement of vagal afferent pathways in the action of ghrelin and des-acy ghrelin was confirmed by c-*Fos* expression in the NTS. I.p. injection of ghrelin significantly increased the density of c-*Fos*-positive cells in the NTS (Figure 2(c)), while i.p. injection of des-acyl ghrelin induced no change in the density of c-*Fos*-positive cells in the NTS compared with vehicle-injected controls [7] (Figure 3(c)). Taken together, these results suggest that peripherally administered des-acyl ghrelin may cross the blood-brain barrier (BBB) and act directly on the brain receptor and disrupt the fasted motility in the antrum (Figure 3(d)).

The centrally administered CRF type 2 receptor antagonist, but not the CRF type 1 receptor antagonist, blocked the effects of centrally and peripherally administered des-acyl ghrelin on gastric motility [7] (Figure 3(b)). Between two CRF receptor subtypes, CRF type 1 receptor is highly involved in anxiety-related behavior and CRF type 2 receptor is involved in regulating food intake and peripheral functions such as gastric acid secretion or gastric emptying. CRF is a relatively selective ligand for CRF type 1 receptor, whereas urocortin 2 is a ligand more selective for CRF type 2 receptor [28, 29]. The density of c-*Fos*-positive cells in the PVN was significantly increased by i.p. injection of des-acyl ghrelin compared to vehicle-injected controls [7] (Figure 3(c)). These data suggest that peripherally administered des-acyl ghrelin may activate neurons in the PVN by crossing the BBB and exert inhibitory effects on the antral motility via CRF type 2 receptor in the brain (Figure 3(d), Table 1).

## 6. Obestatin and Gastroduodenal Motility

Zhang et al. first reported that i.p. injection of obestatin suppressed cumulative food intake, decreased body weight gain, and inhibited gastric emptying and jejunal muscle contraction in mice [2]. Since then, however, the inhibitory effects of obestatin on food intake and gastrointestinal motility have remained controversial [8–13]. Most of the previous studies which showed the negative effects of obestatin on the gastrointestinal motility have only measured the gastric emptying or MMC cycle time as indices for motor activity. In our recent study, for more precise analysis, motor activity in both fed and fasted states was quantified by the %MI, and we measured the time taken to the initiation of phase III-like contractions in the antrum and duodenum of conscious rats [14].

**Figure 4:**
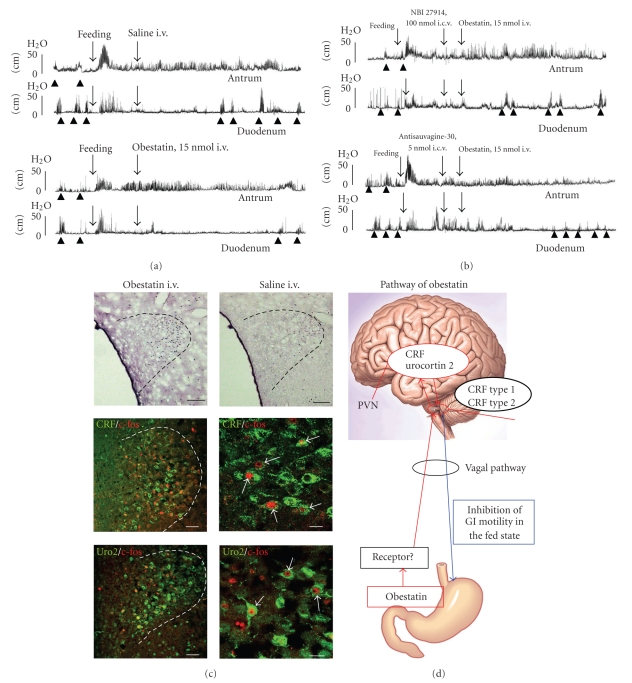
Effects of obestatin on the gastroduodenal motility. (a) Effects of i.v. injection of obestatin on the fed motor activity of the antrum and duodenum. I.v. injection of obestatin prolongs the time between the initiation of phase III-like contractions and injection of obestatin in the duodenum. (b) The elongation of the time between injection of obestatin and initiation of phase III-like contractions in the duodenum induced by i.v. injection of obestatin is reversed by i.c.v. injection of selective CRF type 1 receptor antagonist NBI-27914 and also by selective CRF type 2 receptor antagonist antisauvagine-30. (c) The density of c-*Fos*-positive cells in the PVN is increased by i.v. injection of obestatin compared to saline-injected control. CRF-positive or urocortin 2-positive neurons are overlapped with c-*Fos*-positive neurons in the PVN. (d) Summary diagram of the effects of obestatin on the gastroduodenal motility and brain mechanism mediating its action.

We showed that motor activity in the antrum and duodenum was inhibited when obestatin was given i.v. to conscious rats in the fed state but not when it was given in the fasted state [14]. I.v. injection of obestatin decreased the %MI of fed motility in the antrum and prolonged the time before the return of fasted motility in the duodenum [14] (Figure 4(a)). Such inhibitory actions were the opposite of those obtained with ghrelin [16]. The results showed that the inhibitory action of obestatin appeared 30–90 minutes after i.v. injection [14], which is consistent with the timing of the effects of i.v. injection of ghrelin (~30 minutes) on gastroduodenal motility [16]. I.v. injection of obestatin induced a significant increase in the number of c-*Fos-*positive cells in the PVN compared to saline-injected controls [14] (Figure 4(c)). Immunofluorescence overlap staining showed that the PVN neurons activated by i.v. injection of obestatin contain CRF or urocortin 2 [14] (Figure 4(c)). The involvement of CRF type 1 and type 2 receptors in the action of obestatin on the gastroduodenal motility was examined [14]. Results showed that the inhibitory actions of i.v. injection of obestatin on the motor activities in the antrum and duodenum were blocked by i.c.v. injection of CRF type 1 and type 2 receptor antagonists, suggesting that both types of CRF receptors in the brain may mediate the action of peripherally injected obestatin on gastroduodenal motility [14] (Figure 4(b)). The results showed that vagal afferent nerve blockade by capsaicin reverses the inhibitory effects of obestatin on duodenal motility but does not alter the inhibitory effects of obestatin on antral motility [14]. These results suggest that vagal afferent pathways might be involved partially, but not entirely, in the action of obestatin. Involvement of vagal afferent pathways was confirmed by the finding that the number of c-*Fos*-positive neurons in the NTS was increased by i.v. injection of obestatin [14]. In addition to vagal afferent pathways, it is possible that circulating obestatin acts on brain targets directly by crossing the BBB, because a previous study has shown that there is a rapid influx of i.v.-injected ^125^I-labeled obestatin from the blood to the brain [30]. Therefore the lack of effects of obestatin on antral motility during capsaicin treatment might be explained by direct action of peripherally injected obestatin on brain targets by crossing the BBB, similar to what has been observed for des-acyl ghrelin. We further examined whether obestatin can antagonize the stimulatory effects of ghrelin on gastroduodenal motility [14]. We found that obestatin failed to antagonize the ability of ghrelin either to stimulate the %MI in the antrum or to accelerate the initiation of fasted motility in the duodenum when administrated in the fed state [14]. These results were consistent with previous studies in which obestatin failed to antagonize the ability of ghrelin to stimulate gastric emptying or to shorten the MMC cycle time [8]. 

GPR39 was initially proposed as the receptor for obestatin [2], and GPR39 expression has been detected in peripheral organs such as the duodenum and kidney but not in the pituitary or hypothalamus [4]. However recent publications indicate that obestatin is unlikely to be the endogenous ligand for GPR39 on the basis of a lack of specific binding of obestatin to GPR39 receptor-expressing cells [2, 4, 5, 31]. Nevertheless, although binding of obestatin to the receptor GPR39 remains controversial, the functional effect of obestatin on gastrointestinal motility has been clearly demonstrated in our study.

Our study indicates that obestatin inhibits gastroduodenal motility in the fed state but not in the fasted state of conscious rats. In the brain, CRF- and urocortin 2-containing neurons might be activated by i.v. injection of obestatin, and at the level, CRF type1 and type2 receptors might be involved in the inhibitory action of obestatin on antral and duodenal motility (Figure 4(d), Table 1). Vagal afferent pathways might be involved partially, but not entirely, in these actions of obestatin (Figure 4(d), Table 1).

## 7. Conclusion

Although ghrelin, des-acyl ghrelin, and obestatin are derived from a common prohormone, originating from endocrine cells in the stomach, their roles on the gastrointestinal motility are quite different each other. Ghrelin stimulates the gastroduodenal motility in both fed and fasted states, des-acyl ghrelin inhibits the stomach motility in the fasted state, and obestatin inhibits the gastroduodenal motility in the fed state of animals (Table 1). Different hypothalamic peptides are involved in these actions, NPY Y2 and Y4 receptors may mediate the action of ghrelin, CRF type 2 receptor may mediate the action of des-acyl ghrelin, and CRF type 1 and type 2 receptors may mediate the action of obestatin (Table 1). The regulatory roles of ghrelin, des-acyl ghrelin, and obestatin on the gastrointestinal motility might give us the therapeutic strategies for the functional disorders of the gastrointestinal tracts.

## Figures and Tables

**Table 1:** Summary of the regulatory roles of ghrelin, des-acyl ghrelin and obestatin on the gastroduodenal motility.

	ghrelin		des-acyl ghrelin		obestatin	
	Fasted state	Fed state	Fasted state	Fed state	Fasted state	Fed state
Stomach	↑	↑	↓	—	—	↓
Duodenum	↑	↑	—	—	—	↓
Hypothalamic neuron	NPY		urocortin 2		CRF, urocortin 2	
Brain receptor	Y2, Y4		CRF type 2		CRF type 1, type 2	
Vagal afferent pathway	+		—		+	
